# From learned value to sustained bias: how reward conditioning changes attentional priority

**DOI:** 10.3389/fnhum.2024.1354142

**Published:** 2024-04-03

**Authors:** Kristin N. Meyer, Joseph B. Hopfinger, Elena M. Vidrascu, Charlotte A. Boettiger, Donita L. Robinson, Margaret A. Sheridan

**Affiliations:** ^1^Department of Psychology and Neuroscience, University of North Carolina at Chapel Hill, Chapel Hill, NC, United States; ^2^Bowles Center for Alcohol Studies, University of North Carolina at Chapel Hill, Chapel Hill, NC, United States; ^3^Biomedical Research Imaging Center, University of North Carolina at Chapel Hill, Chapel Hill, NC, United States; ^4^Neuroscience Curriculum, University of North Carolina at Chapel Hill, Chapel Hill, NC, United States; ^5^Department of Psychiatry, University of North Carolina at Chapel Hill School of Medicine, Chapel Hill, NC, United States; ^6^Carolina Population Center, University of North Carolina at Chapel Hill, Chapel Hill, NC, United States; ^7^Frank Porter Graham Child Development Institute, University of North Carolina at Chapel Hill, Chapel Hill, NC, United States

**Keywords:** attention, reward, fMRI, striatum, vmPFC

## Abstract

**Introduction:**

Attentional bias to reward-associated stimuli can occur even when it interferes with goal-driven behavior. One theory posits that dopaminergic signaling in the striatum during reward conditioning leads to changes in visual cortical and parietal representations of the stimulus used, and this, in turn, sustains attentional bias even when reward is discontinued. However, only a few studies have examined neural activity during both rewarded and unrewarded task phases.

**Methods:**

In the current study, participants first completed a reward-conditioning phase, during which responses to certain stimuli were associated with monetary reward. These stimuli were then included as non-predictive cues in a spatial cueing task. Participants underwent functional brain imaging during both task phases.

**Results:**

The results show that striatal activity during the learning phase predicted increased visual cortical and parietal activity and decreased ventro-medial prefrontal cortex activity in response to conditioned stimuli during the test. Striatal activity was also associated with anterior cingulate cortex activation when the reward-conditioned stimulus directed attention away from the target.

**Discussion:**

Our findings suggest that striatal activity during reward conditioning predicts the degree to which reward history biases attention through learning-induced changes in visual and parietal activities.

## Introduction

Reward-associated stimuli continue to distract even when the prospect of reward is removed or devalued when reward-driven distraction is at odds with current task goals or in the presence of a physically salient stimulus (Anderson et al., 2011; Le Pelley et al., 2015; Bourgeois et al., 2017; De Tommaso and Turatto, 2021). These findings cannot be accounted for by models of attention that suggest only two modes of orienting: voluntary goal-directed and involuntary stimulus-driven orienting (Corbetta and Shulman, 2002). A third model (Awh et al., 2012; Failing and Theeuwes, 2018) suggests that signals biasing attention from top-down control, physical salience, and history effects all converge on a priority map, which represents the spatial location most likely to be selected (Itti and Koch, 2001; Theeuwes, 2010; Zelinsky and Bisley, 2015; Failing and Theeuwes, 2018). Reward history appears to exert a strong effect on attentional selection and modulates effects of both voluntary and involuntary orientation (Bourgeois et al., 2017).

A current prevailing theory proposes that striatal dopamine release during reward learning drives changes in visual cortical and parietal representations of stimuli, which biases attentional selection in favor of reward-associated stimuli (Anderson, 2019). Importantly, the current study addresses the untested hypothesis of this model that striatal activity during conditioning is associated with subsequent changes in visual cortical and parietal activities that drive attention. Participants first completed a “reward training phase” during which reward is associated with a stimulus in a visual search task, followed by a “cueing testing phase” during which the previously rewarded stimulus serves as a non-predictive cue in an attention cueing paradigm. If this hypothesis holds, we expect striatal activity during reward training to predict attentional capture by reward and visual and parietal activities in the presence of a task-irrelevant and previously reward-associated stimulus. The second aim of this study is to better characterize interactions between goal-directed attention and reward-driven attention. As such, this study tests the effects of reward history on neural activity during a subsequent attention-cueing paradigm. Since modulations of goal- and reward-driven attention can be additive when directed to the same stimulus (Garcia-Lazaro et al., 2018), this study aims to examine which regions are recruited when goal-driven attention overcomes reward-driven attention. When the reward-conditioned cue is presented alongside a neutral cue, the target may appear in either the location of the neutral cue (“invalid trials”) or the location of the reward-conditioned cue (“valid trials”). By directly contrasting activity for invalid versus valid trials, we can examine which regions support successful deployment of goal-directed *over* reward-driven attention while controlling for visual input from reward-associated stimuli. Top-down attention, which also modulates visual and parietal activity (Li et al., 2004; Buschman and Miller, 2007), must be employed in the presence of a reward-associated distractor, to maintain goal-directed behavior. Current evidence suggests that executive function is inversely related to reward-driven attentional bias (AB; Anderson et al., 2011); however, the brain regions involved in this process remain under explored. The findings from recent research, utilizing the original visual search task (Anderson et al., 2011), suggest the involvement of the value-driven attentional network (VDAN) in processing reward-associated distractors (Kim and Anderson, 2020).

The VDAN, comprised of the caudate tail, early visual cortex, lateral occipital complex (LOC), and the intraparietal sulcus (IPS), plays a role in biasing attention to reward (Anderson, 2017; for reviews see Anderson et al., 2016; Anderson, 2019). It has previously been shown that during the reward training phase prior to measuring attentional bias, reward amplifies stimulus representation in the visual cortex (Serences, 2008; Serences and Saproo, 2010; Itthipuripat et al., 2019), with increased occipital cortex activity, leading to subsequently larger reward-associated distraction effects (van Koningsbruggen et al., 2016). Furthermore, visual cortical activity feeds forward to the parietal cortex, where signals biasing attention converge on the attentional priority map (Zelinsky and Bisley, 2015; Bisley and Mirpour, 2019). One such signal is the presence of reward-associated distractors (Anderson et al., 2014; Barbaro et al., 2017), which further influence the attentional priority map (Failing and Theeuwes, 2018; Todd and Manaligod, 2018; Anderson, 2019). Bourgeois et al. (2022) recently demonstrated that functional connectivity between the dorsal frontoparietal network, the striatum, and the visual cortex, during a rewarded test phase, was affected by both cue validity and reward history. However, no studies have examined neural activity during both a reward conditioning *and* an unrewarded testing phase, limiting inference regarding direct relationships between these processes. As such, this study tests both the effects of reward history on neural activity during the reward training phase and, subsequently, in the cueing testing phase that measures attentional bias.

While there is evidence for likely neural mechanisms underlying the impact of reward conditioning on attention, the current study addresses an important untested link between reward conditioning and later attentional bias. An increase in visual cortical representation of reward could be driven by dopaminergic prediction-error signals in midbrain regions, with the visual cortex being sensitive to both timing and probability of reward receipt (Shuler and Bear, 2006; Arsenault et al., 2013). It has been proposed that projections from the midbrain to the visual cortex modulate early visual activity, which, in turn, modulates activity in the parietal priority map (Anderson, 2019). Consistent with this, previous studies have found a direct relationship between dopamine release and blood oxygen level dependent (BOLD) signal in the visual cortex (Decot et al., 2017; Walton et al., 2021). The caudate has been implicated in learning and sustaining reward-driven attentional biases (Anderson et al., 2016, 2017; Bourgeois et al., 2022), and attentional bias toward reward has been associated with dopamine-modulated connections between the striatum and cortex (Elton et al., 2021). The present study addresses a current gap in the literature by explicitly testing the relationship between neural activity in the caudate during reward conditioning and the subsequent behavioral and neural correlates of attentional bias to the previously rewarded stimulus.

When presented with a rewarding stimulus that no longer serves as a target and instead serves as a distractor, regions in the salience network must communicate with those of the executive network to resolve competing signals on the priority map and guide attentional selection. The anterior cingulate region (ACC) is one such region of the salience network that plays an important role in reactive filtering of distractors and response conflict resolution (MacDonald et al., 2000; De Pisapia and Braver, 2006; Grandjean et al., 2012; Marini et al., 2016) and is therefore expected to be preferentially active for trials when a distractor is located in a different location than the target. In previous investigations, the insula, also a part of the salience network, has been observed to respond preferentially to previously rewarded over unrewarded stimuli (Anderson et al., 2014; Wang et al., 2015; Meyer et al., 2021) and shows a direct relationship with reward-modulated early visual activity (Lee and Shomstein, 2013). Thus, the insula may act as a relay station through which reward history interacts with the frontoparietal control network (Wang et al., 2015; Meyer et al., 2021). Consistent with these observations, it is expected that insula activity will increase when rewarded stimuli are present, specifically for invalid compared with valid trials. Regions in the fronto-parietal attention network that may communicate with the insula include the supplementary motor area (SMA), which may be responsible for initiating voluntary movements when the salience of external stimuli is perceived. The SMA supports aspects of attention, perception, and executive control (Markett et al., 2014; Meyer et al., 2018; Martín-Signes et al., 2021). Therefore, we expect that the activation of the SMA may assist attentional control regions in encoding the timing of cues during the cueing testing phase.

In summary, we hypothesize that striatal activity during reward conditioning will predict sustained changes in parietal and visual cortical activity, and we expect those trials, for which goal- and reward-driven bias compete, will show greater activity in top-down control regions, including the ACC and insula. By collecting neuroimaging data and implementing a task design in which goal-driven and reward-driven bias are aligned on some trials and at odds on others, this study will identify which neural regions differ between converging and competing biases.

## Materials and methods

### Participants and design

Participants were recruited as part of a larger study, investigating the impact of adolescent alcohol history on frontolimbic circuitry and behavioral flexibility. In the parent study, adult participants were recruited into two groups; a control group (no self-reported binge episodes <age 18) and a binge drinking group (≥4 binge episodes <age 18). Most participants in the current study were in the control group (*n =* 19), with only 8 participants in the group that self-reported a history of adolescent binge drinking, which we defined as having 4 or more binge episodes before the age of 18 years (within 2 h: males: ≥5 drinks; women: ≥4 drinks). The aim of the current study is to investigate the effects of reward conditioning on attentional priority and associated neural activity; alcohol use was not a variable of interest. As such, data from a small subsample of participants were used for the analysis of the current study. These participants completed all study procedures required as a part of the larger study. At session 1, participants completed questionnaires and a reward-conditioning training task. At session 2, participants underwent functional magnetic resonance imaging (fMRI) scanning, during which they completed the reward-conditioning task, followed by a modified cueing paradigm using the *previously rewarded* (PR) and *previously unrewarded* (PU) stimuli from the conditioning task.

Participants were *N* = 27 adults aged 22–40 years (*M* = 26.3 years, *SD* = 4.7 years). The selected sample size is consistent with several studies with similar approaches, examining the impact of reward on neural activity in the value-driven attentional control network (Anderson et al., 2014; Anderson, 2017; Kim and Anderson, 2019; Bourgeois et al., 2022). Participants self-reported gender identity (85% women, 15% men), race (15% Asian, 11% Black, 56% white, 15% biracial/ multiracial, and 4% preferred not to respond), and ethnicity (4% Hispanic or Latinx, 85% Not Hispanic or Latinx, and 8% no response). Participants were recruited from print advertisements, flyers, and email listservs. Individuals were excluded from participation for a history of neurological or psychiatric diagnoses, current psychoactive medication, or illicit drug use. We also excluded individuals who were left-handed, color-blind, based on the Ishihara color blindness test (Ishihara, 1973), or who had any fMRI contraindications.

Participants provided informed consent to participate in the study and earned monetary bonuses for task performance. All procedures were approved by the University of North Carolina at Chapel Hill Institutional Review Board.

### Reward-driven attentional bias task

All tasks were programmed using OpenSesame v3.2.8 (Mathôt et al., 2012). Outside the scanner, tasks were displayed on an HP laptop computer, and motor responses were recorded using the keyboard. Inside the fMRI scanner, tasks were projected onto a screen which was viewed via a head coil-mounted mirror, and manual response selection by participants was recorded with a magnetic resonance-compatible button box placed in each hand.

#### Training

**Figure 1:**
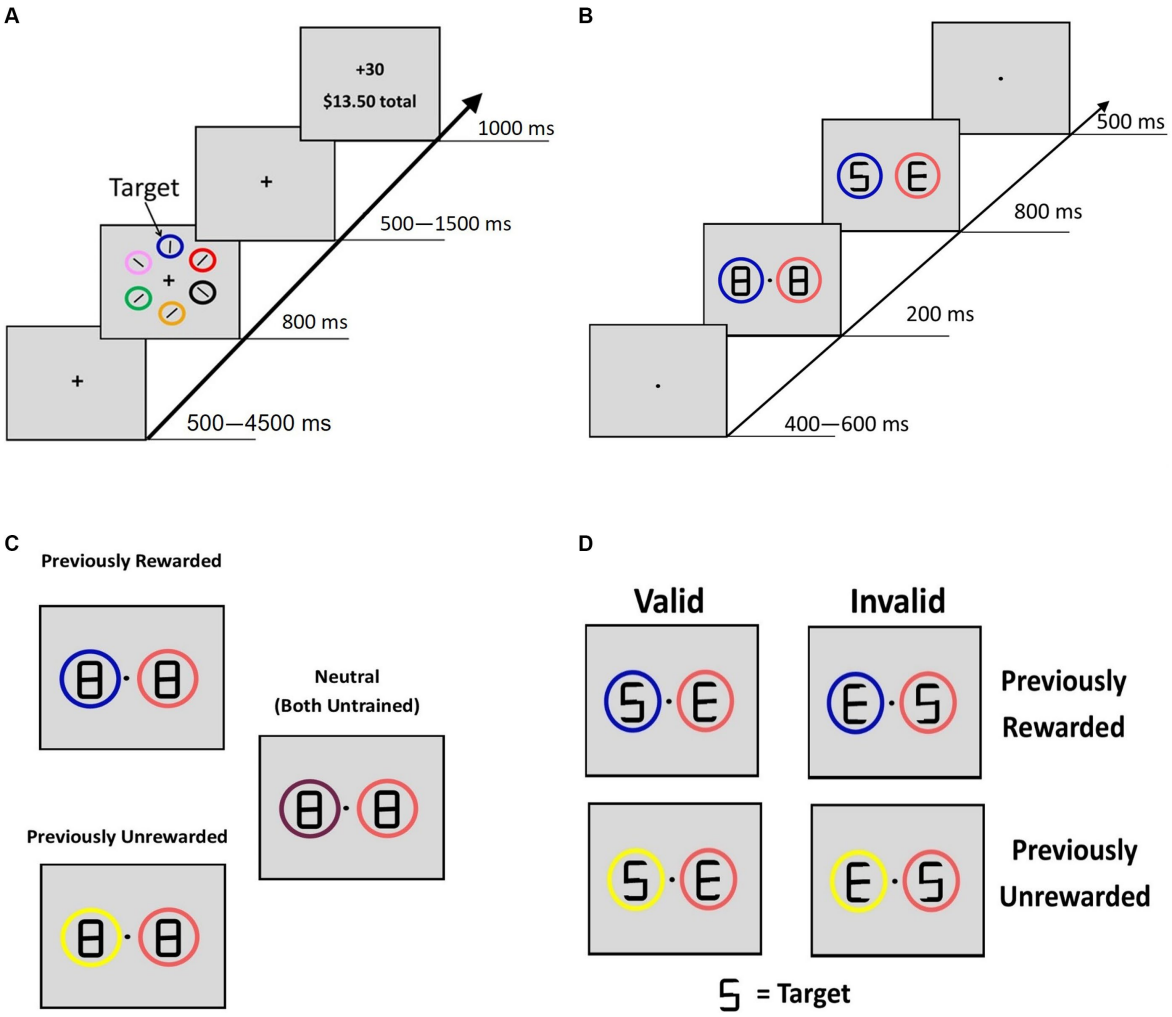
Reward-training and cueing/testing paradigm. **(A)** Adapted from Anderson et al. (2011). Participants were instructed to respond to the orientation of a bar inside the target circle. For the rewarded target circle, 80% of trials were worth 30 cents and 20% were worth no money. For the unrewarded circle, all trials were worth no money. **(B)** Adopted from Failing and Theeuwes (2014). A double cue was displayed for 200 ms with figure-eight premasks, then an offset would reveal target letters for 800 ms. Participants identified whether the letter “S” or the letter “P” was present. Letters “E” and “H” never served as the target letters. **(C)** Description of the 3 classes of cue displays if blue were the previously rewarded color in training, yellow were the previously unrewarded color, and the other colors (peach and maroon) never served as targets during the reward training. **(B)** Example of “valid” and “invalid” trial types for PR and PU trials.

Using a modified training paradigm from the study by Anderson et al. (2011), reward conditioning trials began with a fixation screen (jittered time interval, 500–4,500 ms, *M* = 1,350 ms, SD = 1,062 ms), followed by a search array that consisted of six different colored circles placed at equal intervals around an imaginary circle with a 5° radius from the fixation cross (Figure 1A). In each trial, the rewarded ($0.30/trial, 80% rewarded trials) target circle was either blue or yellow (counterbalanced across participants), and participants were explicitly informed of which color was rewarded. They were also informed that another target circle color was never rewarded. The other colored circles never severed as target circles. To avoid habituation to reward and thus limit reward learning (Mirenowicz and Schultz, 1994; Hollerman and Schultz, 1998), our paradigm implemented a probabilistic reward rather than having 100% prediction certainty. Target location was randomized, and each target circle color was presented equally often. Participants had 800 ms to indicate with a button response (by either their left or right index finger using MR-compatible button boxes) whether the white bar inside the target circle was vertical or horizontal. After the search display, a fixation screen was displayed (jittered time interval, 500–1,500 ms, M = 812 ms, SD = 410 ms) before a feedback screen (1,000 ms) showed the reward amount. The feedback display was blank if participants responded incorrectly and “Slow” if no response was given.

At session 1, participants completed three 120 trial blocks of the training task. At session 2, participants first completed a short refresher. Then, two runs (4 min each) of the training task were performed during fMRI scanning (120 trials total), while the task was projected onto a screen viewed via a head coil-mounted mirror, and participants responded with their left and right index fingers using MR-compatible button boxes.

#### Testing

The cueing task was a modified attention cueing paradigm (Failing and Theeuwes, 2014). The fixation was displayed at the start of each trial (jittered time interval, 500–4,500 ms, *M* = 1,323 ms, SD = 1,061 ms). The cueing display (200 ms; Figure 1B) consisted of two differently colored circles (peach, maroon, yellow, or blue; 3.4° diameter) with figure-eight placeholders (2.3° × 1.1°). The peach and maroon circles were displayed during training but never served as targets, so they served as neutral familiar cues. Thus, these colored circles were viewed a similar number of times during training but they were not the focus of attention, did not elicit a response, and were not associated with reward. After 200 ms, an offset of two line segments in each of the figure-eight premasks revealed the target display, with a letter revealed inside each circle (“S,” “P,” “H,” or “E”). A premask with an offset was used to minimize the exogenous orienting triggered by onsets (Jonides, 1981). One of the two letters was a target (“S” or “P”) and the other was not (“H” or “E”). Participants were instructed to indicate which target letter was present (within 800 ms). For this phase, participants were explicitly informed that the circles were unrelated to the location of the target letter, emphasizing that the color of the circles was task-irrelevant. After a short practice, participants completed three runs (8 min each, 480 total trials) during fMRI scanning. Trials were coded as PR (a *previously rewarded* stimulus plus a neutral stimulus), PU (a *previously unrewarded* stimulus plus a neutral stimulus), or neutral (two neutral stimuli) (Figure 1C); all cues were non-predictive of target location. For PR and PU trials, when the target appeared in the location of the previously trained stimulus, the trial was coded as “valid.” When the target appeared in the location opposite to the PR or PU cue, the trial was coded as “invalid.”

### fMRI data acquisition

Scanning was performed on a 3 T Siemens Prisma Scanner using a 32-channel head coil. T1-weighted multiecho MPRAGE volumes were acquired for coregistration with fMRI images (TR = 2,400 ms, TE = 2.24 ms, flip angle = 8°, field of view = 256 × 256 mm, in-plane voxel size = 0.8 mm^3^). Blood oxygen level dependent (BOLD) signal during functional runs was acquired using a gradient-echo T2*-weighted EPI sequence. In total, 72 slices were acquired in the sagittal plane with a multiband factor of 8, which allowed high spatial (2 × 2 × 2 mm^3^ voxel size) and temporal (TR = 800 ms) resolution (TE = 37 ms, flip angle = 52°, bandwidth = 2,290 Hz/Px, echo spacing = 0.58 ms, field of view = 208 × 208 mm). For the first run of the training task and the first and third runs of the cueing task, data were acquired with an anterior to posterior phase encoding direction; a posterior to anterior direction was implemented for the second run of each task. This technique allowed us to implement fieldmap correction for susceptibility-induced distortions. Before each scan, eight images were acquired and discarded to allow for longitudinal magnetization to reach an equilibrium. The duration of both runs of the training task was ~9 min, the duration of all three runs of the cueing task was ~26 min, and the total duration of the scanning session was 2 h.

### Data processing

#### Behavioral data

We quantified attentional bias effects using inverse efficiency (IE; RT/accuracy), a measure that accounts for speed-accuracy tradeoffs (Townsend and Ashby, 1983). RTs below 100 ms were excluded from further analyses; responses that were too slow (>800 ms) were coded as incorrect, which were consistent with previous study utilizing these paradigms (Anderson et al., 2011; Failing and Theeuwes, 2014). For the training task, a two-way repeated-measures ANOVA compared performance inverse efficiency (IE) for rewarded and unrewarded targets between both sessions. For the testing task, a one-way repeated measures ANOVA comparing performance (IE) for invalid PR, neutral, and valid PR trials assessed attentional bias, including facilitated capture for the valid trials and impaired disengagement for the invalid trials. A follow-up one-way repeated measures ANOVA was conducted for the PU trials to determine whether attentional bias is specific to reward or due to general experience. Similar analyses performed on RT and accuracy independently are reported in Supplementary material.

##### Training and testing phase relationships

Activity within the striatum during the training task for rewarded>unrewarded correct trials was extracted for each subject individually using FSL’s feat query function. The ROI sphere was created by drawing a 4-mm sphere around the peak activation within the right caudate observed at the group level (coordinates: *x* = 8, *y* = 18, and *z* = 4, Supplementary Table S2). Then, a group-level GLM was constructed in which the extracted beta weight values of striatal activity from the training task (i.e., predictor variable) were associated with neural activity in the whole brain during the testing task. Finally, to examine whether striatal activity predicts attentional bias to reward in the testing task, we used a non-parametric Spearman correlation between striatal activity during the training phase and attentional bias on PR trials during the testing task (Rousselet and Pernet, 2012).

#### Imaging data

##### Pre- and post-processing

In this manuscript, the results come from processing performed using *fMRIPrep* 1.3.2 (Esteban et al., 2018, 2019), which is based on *Nipype* 1.1.9 (Gorgolewski et al., 2011) and *clpipe* 1.6.0 (Asciutto et al., 2023). The description of the pre-processing steps was automatically generated by *fMRIPrep* software, and the full detail is included in Supplementary material. (for more details see Lanczos, 1964; Cox and Hyde, 1997; Dale et al., 1999; Zhang et al., 2001; Jenkinson et al., 2002; Avants et al., 2008; Greve and Fischl, 2009; Shin et al., 2010; Abraham et al., 2014; Power et al., 2014; Klein et al., 2017; Cortese et al., 2021).

For anatomical preprocessing, each subject’s T1-weighted (T1w) image was corrected for intensity non-uniformity (Tustison et al., 2010), skull-stripped, and spatially normalized to the *MNI* Template (Fonov et al., 2009). A T1w reference map was computed after registration of two T1w images. Brain surfaces were reconstructed before estimating a brain mask used for brain extraction of both the T1w template and volume, which was used to perform brain tissue segmentation of the cerebrospinal fluid and white matter and gray matter. For functional preprocessing, a reference volume and its skull-stripped version were generated prior to correcting for susceptibility distortions using volumes in both phase encoding directions. An unwarped BOLD reference was used to co-register with the T1w reference map (9 degrees of freedom), and the BOLD runs were slice-time corrected. Three confounding variables (framewise displacement, the derivative of root-mean-square variance over voxels, and physiological regressors) were calculated using the BOLD time-series, and six principal components were calculated from the runs in native space. Post-processing consisted of spatially smoothing the data (4 mm FWHM kernel) prior to being inspected for artifacts using a framewise displacement (FD) motion threshold of 0.9 mm. Motion outliers exceeding 0.9 mm, in addition to six rigid body motion regressors and their first temporal derivative (12 motion regressors total), were included as regressors of non-interest for in-person level models. There were no individual runs where more than 20% of the time points were excluded due to motion; thus, no entire runs were excluded. Within the cueing task, three participants had one run of data that could not be included in analysis due to other technical difficulties; only two runs of cueing task data were included for these participants.

##### General linear model estimation

Two general linear models (GLMs), one for the training phase and another one for the testing phase, were used to estimate the effects of task and control for the effects of non-interest. For the training phase, the GLM included covariates for trials with correct responses when the rewarded and unrewarded stimuli were present. Trials with incorrect responses, and trials with the feedback screen, were also included as covariates in the model. For the testing phase, the model included covariates for trials describing the type (neutral, PR valid/invalid, and PU valid/invalid) and target location (right/left) for accurate trials and trials with incorrect responses. These 10 covariates were as follows: (1) neutral trials, (2) PR valid target left, (3) PR valid target right, (4) PR invalid target left, (5) PR invalid target right, (6) PU valid target left, (7) PU valid target right, (8) PU invalid target left, (9) PU invalid target right, and (10) incorrect trials. For both models, six rigid body motion regressors and their first derivative (12 total), and outlier regressors (volumes exceeding FD of 0.9 mm), were included as covariates of non-interest.

##### Group-level analysis

Group-level mixed-effect statistical analyses were implemented in FSL FEAT with FLAME1 (Woolrich et al., 2004; Eklund et al., 2016). Analysis of functional images focused on isolating the BOLD signal in the value-driven attention network during reward learning by contrasting rewarded to unrewarded trials. All results were thresholded using a voxel-wise *Z*-statistic threshold (*Z* = 2.3) and a cluster threshold (*p* = 0.05), to effectively decrease the rate of false-positive findings (Woolrich et al., 2004; Eklund et al., 2016).

##### ROI analysis

Regions of the dorsal attentional control network, including bilateral parietal regions and the supplementary motor area (SMA), were identified by creating a binary mask from the three clusters that reached the threshold when comparing neutral trials with implicit baseline. For the ROI analysis, neutral trials were used in the identification of ROI only and was not included in the analysis following extraction. This approach using task-related activity to define our ROIs was done so that we could remain neutral to later analysis (i.e., looking at PR activity) while increasing sensitivity. After the ROIs were defined, activation values from regions with significant clusters were extracted from the mask for the PR and PU trials relative to baseline using FSL’s feat query.

## Results

### Behavioral results

#### Reward training

A 2 × 2 repeated-measures ANOVA (within-subjects factors: reward status and session) revealed both a main effect of reward type [*F*(1, 26) = 56.44, *p* < 0.001, *η*^2^ = 0.685] and a reward by session interaction [*F*(1, 26) = 5.70, *p* = 0.025, *η*^2^ = 0.180] on inverse efficiency (IE). The results from pairwise comparisons showed that participants performed better on trials with the rewarded versus unrewarded circle (*M*_rewarded_-*M*_unrewarded_ = −168.829, *p < 0.*001, 95%CI [−215.022–122.636]), and these effects were stronger in session 2 compared with session 1 (M1-M2 = –201.750, *p <* 0.001, 95%CI [−269.505–133.994]). This suggests that while participants performed better for the rewarded color across both sessions, the effect of reward on performance was larger in the second session (Supplementary Table S1).

#### Testing phase/attention cueing paradigm

**Figure 2:**
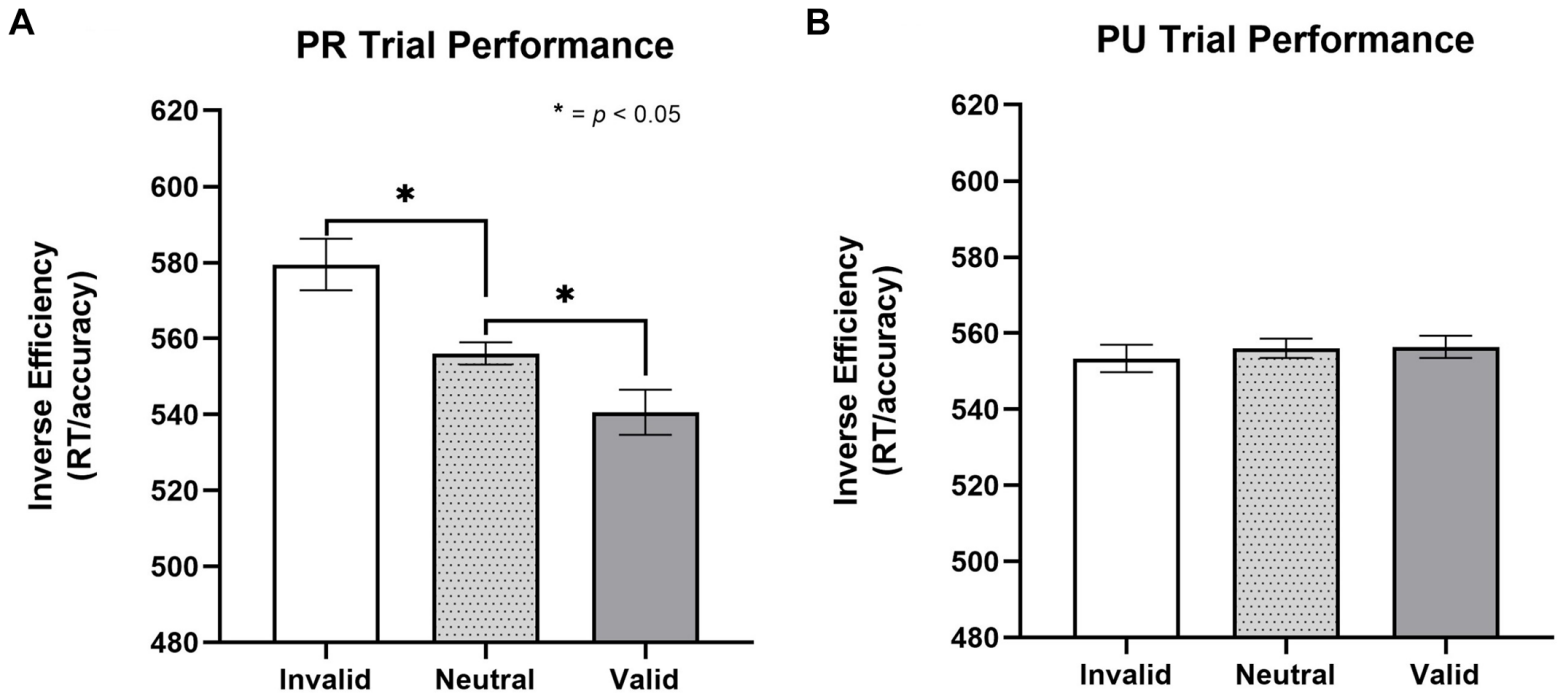
Reward training and cueing/testing task whole brain results. **(A)** Whole brain activation for Rewarded stimulus trials > Unrewarded stimulus trials. Blue arrow indicates the striatal region (caudate), from which beta weights were extracted for further analyses. **(B)** Whole brain activation for all trial types compared to an implicit baseline. **(C)** Whole brain activation for PR trials > Neutral trials, demonstrating visual cortical and attentional control regions with preferential activity to PR cues.

To test for a linear-orienting effect in performance on PR trials, a repeated-measures one-way ANOVA was conducted with trial type (invalid, neutral, and valid) as the within-subjects factor. There was a significant effect of trial type [*F*(1, 29) = 8.58, *p* = 0.003, *η*^2^ = 0.248; Figure 2A]. Paired *t*-tests revealed a significant effect of both facilitated *“capture”* to the PR cue (valid better than neutral), *t*(26) = 2.41, *p* = 0.023, *d* = 0.464, and impaired “*disengagement*” from the PR cue (invalid worse than neutral), *t*(26) = 2.73, *p* = 0.011, *d* = 0.525 (*M*_Valid_ = 541, SD_Valid_ = 72; *M*_Neutral_ = 556, SD_Neutral_ = 79; *M*_Invalid_ = 579, SD_Valid_ = 111).

To investigate whether the linear orienting effect was unique to the history of reward (i.e., value-specific), we examined whether a similar effect was observed for previously unrewarded (PU) cues. This involved conducting a one-way, repeated-measures ANOVA with trial type (invalid, valid, neutral) as the within-subjects factor. There was no effect of trial type on inverse efficiency, *p* = 0.819 (Figure 2B); as such, the components of attentional bias (i.e., capture and disengagement) to PU cues were not further probed (*M*_Valid_ = 556, SD_Valid_ = 88; *M*_Neutral_ = 556, SD_Neutral_ = 79; *M*_Invalid_ = 553, SD_Valid_ = 75, *p* = 0.819).

### Neural activity results

#### Training phase

**Figure 3:**
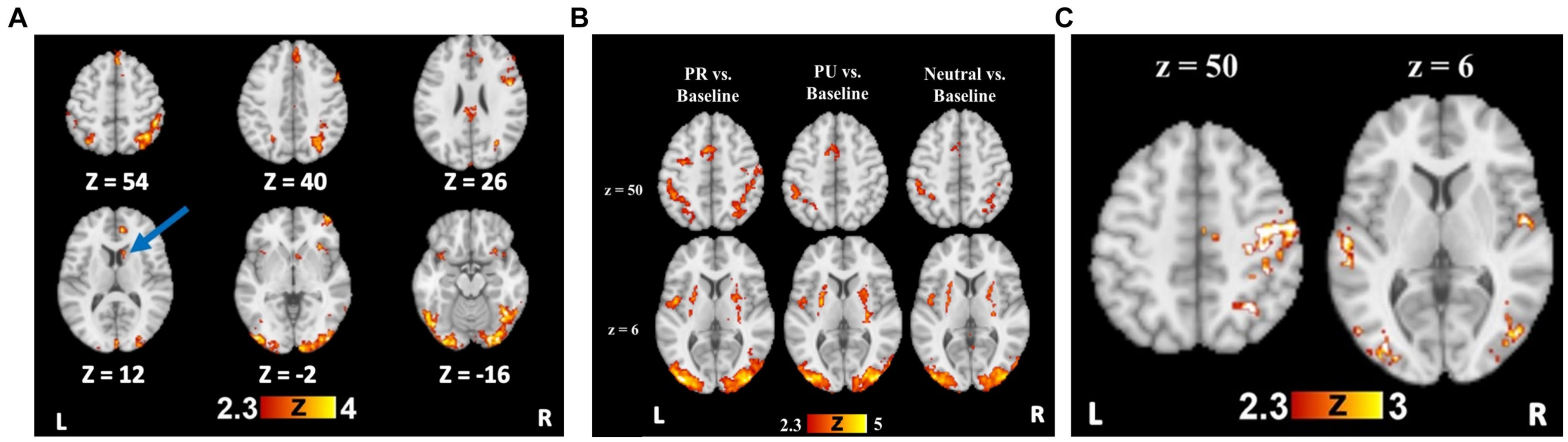
Effect of trial type on inverse efficiency for previously rewarded and non-rewarded trials. **(A)** Orienting effects to PR cues on the cueing task for inverse efficiency (*M*_Valid_ = 541, SD_Valid_ = 72; *M*_Neutral_ = 556, SD_Neutral_ = 79; *M*_Invalid_ = 579, SD_Valid_ = 111). A significant linear orienting effect was found for PR trials, characterized both by facilitated capture on PR valid trials and impaired disengagement on PR invalid trials. **(B)** No evidence (*p* = 0.819) of orienting effects to PU cues (*M*_Valid_ = 556, SD_Valid_ = 88; *M*_Neutral_ = 556, SD_Neutral_ = 79; *M*_Invalid_ = 553, SD_Valid_ = 75, *p* = 0.819). Error bars are within subjects 95% confidence intervals (Cousineau, 2005). Inverse efficiency = RT/accuracy. PR, previously rewarded; PU, previously unrewarded.

To test reward-specific effects, we examined group maps with the rewarded>unrewarded stimulus contrast, which demonstrated a robust effect of reward conditioning on neural activity. Relative to unrewarded visual search targets, rewarded targets elicited greater BOLD signal in bilateral visual cortical regions, bilateral parietal cortex, and prefrontal regions (Supplementary Table S2 and Figure 3A).

#### Testing phase

A history of reward was associated with greater activity in regions including the right superior parietal lobule (SPL), SMA, bilateral occipital fusiform gyri, and LOC for the PR > Neutral contrast (Supplementary Table S3 and Figure 3C). For the PU > Neutral contrast, no activity was observed in parietal regions or visual cortex (Supplementary Table S3). Only a cluster in the precentral gyrus showed greater activity for PR relative to PU trials (Supplementary Table S3).

**Figure 4:**
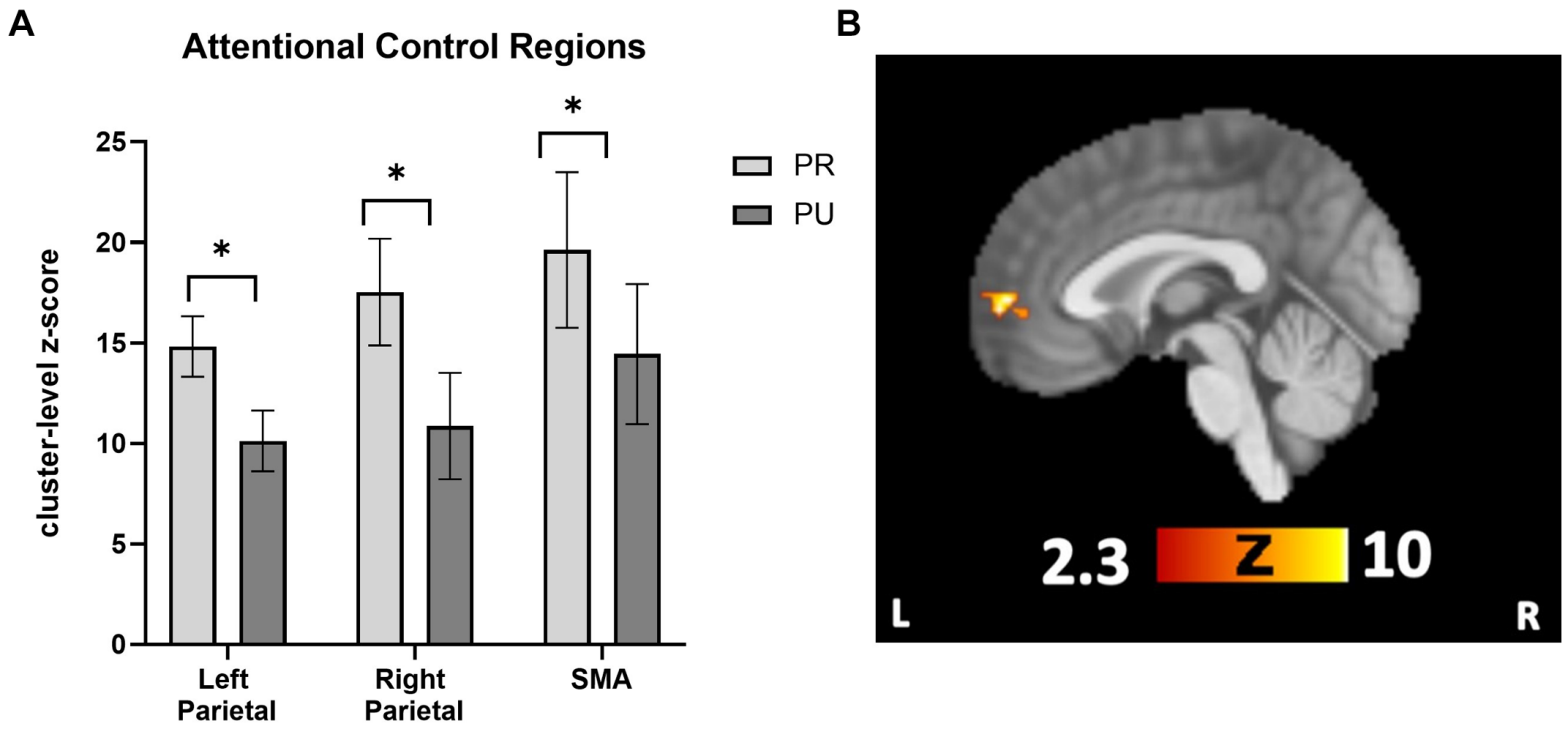
Cueing/testing task attentional control roi and conjunction overlay results. **(A)** Results revealed a significant main effect of reward conditioning on neural activity (displayed as cluster-level *z*-score on *y*-axis) activity across attentional control regions for PR trials relative to PU trials (*p* < 0.001, η2 = 0.664). Activity across ROIs was significantly greater for PR versus PU trials (PR-PU, 5.32; *p* = 0.009; CI, [1.42, 9.21]). There were no overall differences in activation between ROI’s (*p* = 0.451) nor were there qualifying interactions between the effect of reward history and region (*p* = 0.755). **(B)** Activity in the vmPFC during PR < PU (and Neutral) of Cueing/Testing task. Conjunction overlay demonstrating deactivation in vmPFC for PR trials relative to both Neutral trials and PU trials (sagittal slice displayed at *x* = 0). ROI, region of interest; SMA, supplementary motor area. *p<0.05, FWE corrected.

To follow-up on *a priori* hypotheses that reward history would be associated with increased activity in attentional control regions, ROI analyses were conducted in attentional control regions (bilateral parietal cortex and SMA). Activity in parietal and SMA regions for the contrast of neutral>implicit baseline was used to define the location of the ROI. These ROIs were queried separately for PR > implicit baseline and PU > implicit baseline. The SMA and bilateral parietal regions were created by creating a binary mask from the three clusters in Neutral > implicit baseline that corresponded to these regions. A 2 × 3 repeated measures ANOVA with trial type (PR, PU trials) and ROI (right parietal, left parietal, and SMA) revealed a main effect of reward history, *F*(1, 26) = 51.304, *p* < 0.001, η^2^ = 0.664, such that PR trials showed more activation than PU trials (Figure 4A). Pairwise comparisons revealed a mean difference in neural activity of 5.32 (*p* = 0.009, CI = [1.42, 9.21]) for PR compared with PU trials. There were neither overall differences in activation among ROIs (*p* = 0.451) nor was there any interaction effect of reward history and region (*p* = 0.755).

Next, we tested whether any regions showed a relative *deactivation* to PR versus PU or Neutral stimuli. For the contrast PU > PR, whole-brain results revealed one significant cluster in the vmPFC (Supplementary Table S3). For Neutral>PR, there were significant clusters in the vmPFC, middle frontal gyrus, right LOC, and angular gyrus (Supplementary Table S3). To confirm overlapping activity in the vmPFC for both contrasts, a conjunction overlay was performed. The results revealed one overlapping area in the vmPFC, which was active for both the PU > PR and Neutral>PR analyses (105 voxel cluster size; local maximum *x* = −2, *y* = 58, *z* = 2; Figure 4B). There were no areas which were significant for the Neutral > PU contrast.

Finally, to test our hypothesis that frontal control regions, including the ACC and insula, would be recruited when goal-driven and reward-driven attention are in conflict, we assessed neural activity in response to PR invalid trials relative to PR valid trials, neutral trials, or PU invalid trials. Contrary to our prediction, for the contrasts PR Invalid>PU Invalid and PR Invalid>Neutral, greater activity was found in the lateral visual cortex and parietal regions (Supplementary Table S4) but not in the ACC or insula. For the PR Invalid>PR Valid contrast, no clusters reached significance.

### Training and testing phase

**Figure 5:**
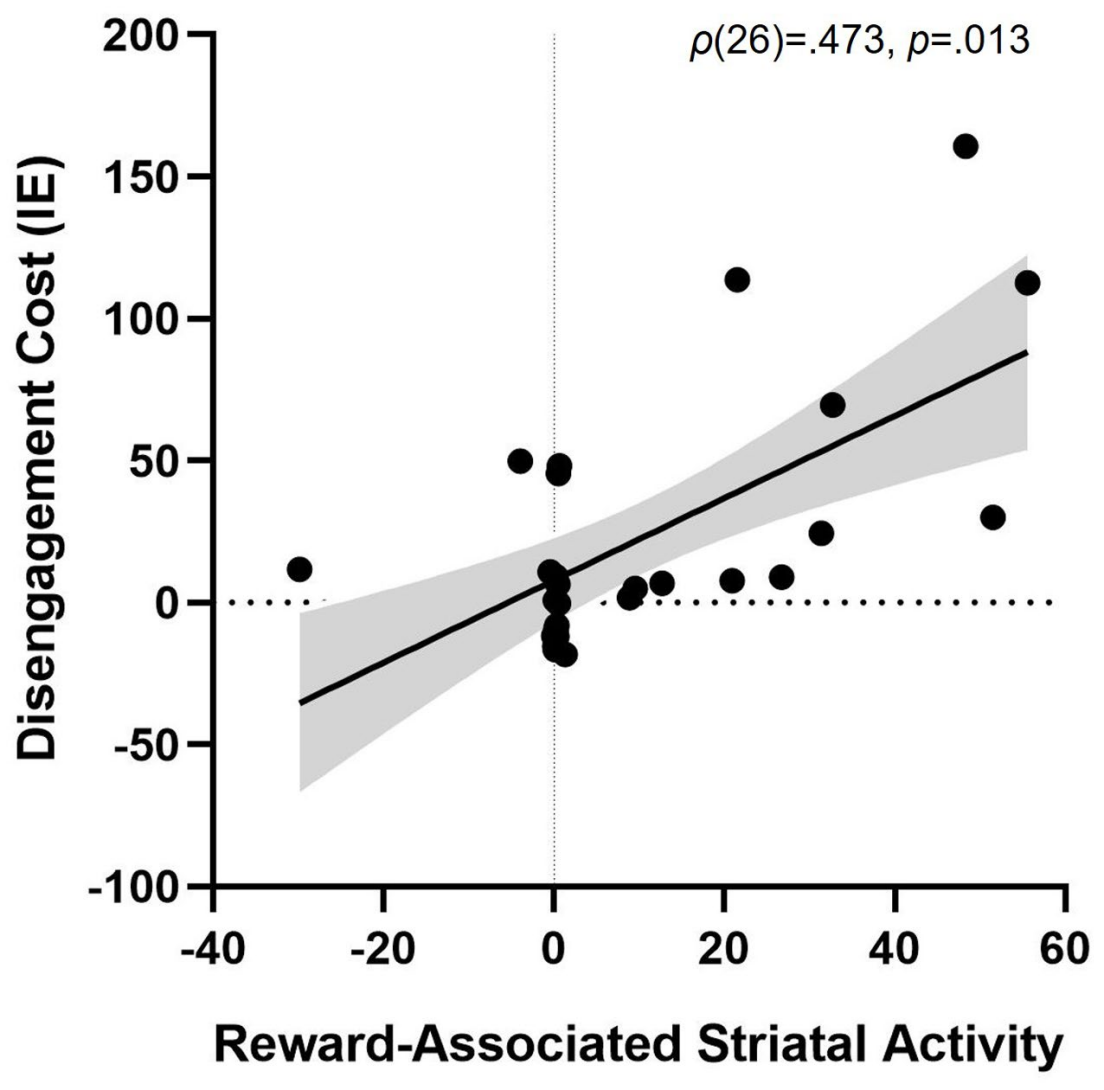
Association between striatal activity during reward conditioning and attentional bias. More striatal activity for trials with a Rewarded stimulus > trials with an Unrewarded stimulus during training predicting disengagement cost of having a reward-associated distractor present during test (PR Invalid relative to Neutral performance), *ρ*(26) = 0.473, *p* = 0.013. Striatal activity is represented as the beta weight values during the contrast rewarded > unrewarded trials in the training phase. Shaded grey area represents the 95% confidence bands. Disengagement cost = [Invalid IE – Neutral IE]. IE, inverse efficiency (RT/accuracy).

Striatal activity during the *training task* (Rewarded > Unrewarded trials) was associated with greater disengagement cost in the *testing task* (i.e., worse performance on PR Invalid relative to Neutral trials), *ρ*(26) = 0.473, *p* = 0.013 (Figure 5). We also found positive associations between striatal activity and both overall attentional bias to reward (*p* = 0.479) and attentional capture by reward-conditioned stimuli (*p* = 0.464), although no correlation approached significance.

To examine whether striatal activity during the training task correlated with patterns of neural activity during the testing task, we looked at contrasts with and without considering cue validity. Striatal activity predicted more activity in visual and parietal areas for the contrast PR > Neutral (Supplementary Table S5). No clusters reached significance for the PR > PU or Neutral>PR comparisons. However, for PU > PR, more striatal activity was correlated with activity in the vmPFC, indicating *less* activity in the vmPFC in response to PR versus PU trials. When considering cue validity, for PR Valid > Neutral, more striatal activity was associated with more activation in visual areas, the angular gyrus, and the motor cortex (Supplementary Table S5). No clusters reached the threshold for the contrast PR Valid > PU Valid. However, more striatal activity predicted relatively greater activation in the ACC and supramarginal gyrus for the PR Invalid > Neutral and PR Invalid > PU Invalid contrasts.

## Discussion

This study examined how reward conditioning leads to attentional bias toward a previously rewarded stimulus. The results demonstrate that attention is biased in response to reward-conditioned stimuli via increased neural activity in visual and attentional control regions. Furthermore, this study is the first of its type to test the relationship between striatal activity during reward conditioning (i.e., training task) and neural activity during an unrewarded testing task. Our results demonstrated that striatal activity during reward conditioning predicts both visual cortical activity during the testing task and behavioral indices of the degree to which participants bias attention to the reward-conditioned stimulus. Finally, we sought to evaluate potential differences in neural activity when reward- and goal-driven attentional control align or conflict. We hypothesized that there would be increased activation of frontal control regions for trials where bottom-up and top-down attentional control conflict. However, our results indicate largely overlapping activity in the value-driven attention network (VDAN) for both forms of attentional control, specifically in the ventromedial prefrontal cortex (vmPFC).Our pattern of results suggests that participants may resolve conflict arising as a result of reward history through deactivation of the vmPFC, potentially signaling a devaluation of the previously rewarded stimulus.

### Reward conditioning

Evidence of successful learning was demonstrated by better performance for reward-conditioned targets, which elicited activity in the VDAN, including the LOC, parietal cortex, caudate, and early visual cortex, consistent with previous studies (Anderson, 2017; Antono et al., 2023; for reviews see Anderson et al., 2016; Anderson, 2021). Rewarded targets were also associated with greater activity in the anterior insula, a region that is associated with reward learning (Liu et al., 2011) and value-driven attentional selection (Wang et al., 2015). Furthermore, we found that regions which support cognitive control, including the ACC, IFG, and ventrolateral prefrontal cortex (Cole and Schneider, 2007; Braver et al., 2009; Niendam et al., 2012; Deng et al., 2018), were preferentially active in the presence of rewarded targets, which is less commonly observed in value-driven attention paradigms. This may be because participants in the current study were provided explicit knowledge of which stimulus conferred higher reward prior to starting the task. As such, participants may have additionally employed top-down strategies, relying on explicit information about the availability of reward (Daw et al., 2005; Feifei et al., 2018). Another possibility is that a few participants with a history of adolescent alcohol use may have differential activity in executive and salience network regions, thereby requiring greater activity in these regions when presented with rewarding stimuli. Investigating the effects of alcohol use during this critical development period was outside the scope of the current study but should be investigated further.

### Reward history effects on AB and underlying neural circuitry

Despite the non-predictive nature of the cues after reward conditioning, participants still exhibited attentional bias to the previously rewarded cue. Replicating previous findings (Failing and Theeuwes, 2014), this effect was reward-specific, as we detected no attentional bias to the familiar but previously unrewarded cue. Consistent with previous studies, the degree to which a participant’s striatal activity selectively increased for rewarded stimuli correlated with the degree to which they demonstrated attentional bias to the previously rewarded stimulus (Anderson et al., 2016, 2017; Meffert et al., 2018). This is an important link to test the prevailing current theory that dopaminergic signals in the striatum during reward learning drive changes in visual cortical and parietal representations of stimuli, which, in turn, leads to biased attentional selection (Anderson, 2019).

While previous studies have demonstrated that striatal activity amplifies visual cortical and parietal activity *during* learning (Hickey and Peelen, 2015, 2017; Anderson, 2017; Barbaro et al., 2017), this study is the first to provide direct evidence that striatal activity during learning predicts *sustained* changes in parietal and visual cortical representation, even after the removal of reward receipt. Activation within the frontoparietal attention network (Corbetta and Shulman, 2002), particularly in the bilateral parietal cortex and supplementary motor area (SMA), and the visual cortex, was observed across all trial types. As hypothesized, previously rewarded cues were associated with relatively greater activity in these regions compared with neutral, familiar cues. While differences in these regions between previously rewarded and previously unrewarded cues were not observed at the whole-brain level, our results do demonstrate reward-specific effects when activity specifically in these regions, were queried. This finding supports previous models, suggesting that reward history effects converge on the parietal cortex (Failing and Theeuwes, 2018; Anderson, 2019; Bourgeois et al., 2022), and extends them to provide support for reward history effects in the SMA, which is implicated in both voluntary and involuntary attention (Hopfinger et al., 2000; Corbetta and Shulman, 2002; Meyer et al., 2018). This suggests a level of plasticity that is sensitive to reward learning and persists in unrewarded contexts.

Our results also identified the activation of differential ventromedial prefrontal cortex (vmPFC) across reward conditions and sensitivity. In trials that included a previously reward-associated stimulus (in contrast to previously unrewarded cues), we observed a relative deactivation in the vmPFC, which was greatest in individuals who had the strongest striatal response to reward-associated stimuli during conditioning. Previous studies have implicated the role of the vmPFC in supporting behavioral flexibility in the context of changing reward contingencies (Bechara et al., 2000; Fellows and Farah, 2003; Gläscher et al., 2009; Davidow et al., 2019). This could, in part, be due to the role of the vmPFC in value representation, wherein vmPFC activity increases as the value of rewards increases (McClure et al., 2004; Lim et al., 2011). Although this mechanism conflicts with our current finding of a decrease in vmPFC activity, it may reflect a compensatory “updating” of value in a novel, unrewarded context. Specifically, while the absolute value in the cueing task is equal across stimuli, the relative change in value is negative for the previously rewarded stimulus as compared with the previously unrewarded or neutral stimuli. This interpretation is consistent with the evidence that decreases in vmPFC activity are evident after a rewarded stimulus is no longer rewarded (Zhang et al., 2016) and warrant further investigation.

### Interactions between goal-directed and reward-driven attention effects

Another aim of this study was to clarify the neural mechanism through which reward history and goal-directed top-down attention interact. We expected that more activity in frontal control regions would be associated with PR invalid relative to PR valid trials, to override attentional hold by reward-related distractors. Surprisingly, no clusters of voxels reached significance. However, we did find that more striatal activity during rewarded trials in the training task predicted greater activation in the ACC for PR Invalid relative to Neutral trials or PU Invalid trials in the testing task. This suggests that recruitment of the ACC, which aids in the processing of rewarding value of stimuli, might communicate more with self-control regions among individuals who exhibited the greatest disengagement failure. Furthermore, both reward- and goal-driven attention effects might be interacting within the same attention network, as we observed overlapping activation of the ventro-medial prefrontal cortex. Indeed, whole-brain analyses are not often sensitive to detect differences within the frontoparietal network, despite evidence on diverging top-down and bottom-up influences on attentional selection (Corbetta and Shulman, 2002; Buschman and Miller, 2007; Meyer et al., 2018; Bowling et al., 2020). It may be the case, however, that less activity in the vmPFC in response to PR Invalid trials is the result of less communication with frontal control regions, such as the dorsolateral prefrontal cortex, and these individuals exhibit less goal-directed behavior (Hare et al., 2009). Additionally, it is possible that the use of a multiband factor of 8 in acquiring functional scans resulted in poor signal-to-noise in subcortical regions that might otherwise have shown activation differences; however, it is unclear whether this is a concern with task-based scans and not just resting-state scans (Risk et al., 2021). Relatedly, our small sample size might not have afforded enough power to detect existing differences in activation.

Recent research has highlighted separable effects of reward history, facilitating the initial capture of attention versus impairing the latter ability to disengage attention (Meyer et al., 2020; Watson et al., 2020; Zhuang et al., 2021). In this study, striatal activity during reward conditioning predicted the level of disengagement failure from a previously rewarded cue, but not the degree of attentional capture when redirecting attention was not necessary. For valid trials, more striatal activity was associated with greater subsequent activity in the early visual cortex, occipital fusiform gyrus, and the angular gyrus, a region implicated in spatial attention (Chambers et al., 2004; Studer et al., 2014). Individuals with the highest striatal response showed greater anterior cingulate cortex (ACC) activity when attention needed to be disengaged, agreeing with the findings that disengaging from a distractor requires reactive attentional control (Geng, 2014), and that the ACC supports this reactive filtering of distractors (Marini et al., 2016) and disengagement from emotionally salient stimuli (Cisler and Koster, 2010). Taken together, these results suggest that striatal activity during reward conditioning increases visual cortical representation of the previously rewarded stimulus, and that a greater degree of processing in the ACC is necessary to successfully disengage attention from this stimulus and redirect attention toward current task goals. This study provides exciting new evidence that differentiates the relative impact of reward-associated neural activity during learning on later-facilitated initial orienting and impaired suppression of responses to stimuli with a reward history.

### Limitations and future directions

While this study provides novel insight into the mechanisms by which reward history and goal-driven effects interact within the frontoparietal attention network, there are limitations and future avenues to consider for research. First, while many elements of our results are consistent with the effects observed being driven by reward history, it is important to note that we did not observe differences within the value-driven attention network for previously rewarded versus previously unrewarded stimuli when performing whole brain analysis (with stringent corrections). It is possible that target history may be contributing to the differences we observed between the previously rewarded and neutral cues. However, we do not believe that target history can explain the constellation of our results, as we do observe reward-specific differences between the previously rewarded and previously unrewarded cues in behavior, and we observe significant differences in neural activity between those conditions when utilizing analysis of a more sensitive region of interest in just our *a priori* regions of interest within the value-driven attention network. Nevertheless, it will be important for future studies to further disentangle reward history and target history effects within the attention network. Furthermore, our sample size may have impacted the ability to detect small effects, which may, in turn, have impacted our ability to detect differences in neural activity between disengagement failure and facilitated capture.

Future study may benefit from exploring differences in functional connectivity, or the timing of neuronal firing between brain regions, as has been utilized in examining differences in voluntary and involuntary attention (Buschman and Miller, 2007; Bowling et al., 2020). Relatedly, our findings provide implications for the roles of the vmPFC and ACC as neural substrates recruited to resolve discrepancies between goal-directed and reward-driven attention; however, further study is needed to clarify the mechanisms by which they may support such conflict resolution. Specifically, this study was not designed to test how activity in the vmPFC may change across time in service of updating reward-related relevance. Although we found decreased activity in the vmPFC during trials in which a previously rewarded stimulus was present, it could be the case that vmPFC activity was initially increased and then decreased in activity as reward value was updated. Exploring this activity over time may elucidate how quickly updating reward-related relevance is reflected by vmPFC activity among participants who display greater striatal activity during reward conditioning. This could provide insight into individual differences in sensitivity to reward and subsequent attentional bias toward non-drug reward. Given evidence showing that increased attentional bias has been previously associated with increased craving among adult social drinkers (Manchery et al., 2017), investigating dynamic activity in the vmPFC, including functional connectivity with frontal control regions, could inform whether vmPFC activity could be used as a biomarker to identify individuals at risk for developing alcohol or substance use disorder. Greater self-control related to optimal decision-making is supported by increased activity in the vmPFC and dorsolateral prefrontal cortex, a region of the executive network that is important for executing goal-directed behavior (Hare et al., 2009). As such, exploring whether vmPFC and ACC activities drive changes in behavior (e.g., reduced attentional bias *to* reward or faster disengagement *from* rewarded stimuli) is not within the scope of this study and warrants further investigation.

## Conclusion

This study served to fill important gaps in our knowledge regarding the mechanisms through which reward history leads to distraction. To the best of our knowledge, this study is the first to demonstrate a direct relationship between striatal activity during reward conditioning and increased visual cortical and parietal activity in a subsequent attention task without the prospect for reward receipt. More specifically, caudate activity during reward conditioning predicted the degree of disengagement failure on a subsequent task, involving a stimulus with a history of reward conditioning. This study provides new evidence that reward history of a stimulus is associated with relative deactivation in the vmPFC when encountered in a task without reward, which is relative to stimuli that have no history of reward conditioning. Collectively, these findings indicate that reward history influences perceptual processing by inducing alterations in visual and parietal activity. Overcoming this bias requires reactive top-down suppression of the previously rewarded stimulus and a prolonged value-updating process to restore the stimulus value as neutral.

The findings from our study contribute to the body of literature exploring the neural mechanisms involved in attentional bias, potentially informing preventive approaches targeting individuals vulnerable to developing alcohol or substance use disorders, as well as other mental health conditions. Attentional bias has been implicated not only in addiction (Anderson et al., 2013; Field et al., 2014) but also in psychopathology, where difficulty lies in controlling salient external stimuli, such as obesity (Field et al., 2016; Anderson, 2021). It would also be worth exploring whether heavy alcohol use during adolescence, when frontal control regions are expected to significantly mature, impairs behavioral flexibility in adulthood. Although our study sample consisted of less than half of the adult participants having self-reported a history of binge drinking alcohol before the age of 18 years, it is possible that their drinking history might have influenced, to some extent, the degree of attentional bias observed and should be probed further. Moreover, it would be interesting for future studies to look at the associations between attentional bias toward non-drug reward and interoceptive cues that guide attention, such as anxiety and depression. Recent research has demonstrated that attentional bias toward negative (external) stimuli increases with trait anxiety, so discovering novel pharmacological therapeutics or medical device treatments such as brain stimulation could be beneficial toward the prevention of many disorders (Veerapa et al., 2020; Anderson, 2021). Furthermore, attentional bias toward alcohol stimuli has been associated with craving among adult social drinkers (Manchery et al., 2017), which warrants further investigation in attentional bias toward non-drug reward as an intermediate phenotype for addiction and other disorders.

## Data availability statement

The raw data supporting the conclusions of this article will be made available by the authors, without undue reservation.

## Ethics statement

The studies involving humans were approved by University of North Carolina at Chapel Hill Institutional Review Board. The studies were conducted in accordance with the local legislation and institutional requirements. The participants provided their written informed consent to participate in this study.

## Author contributions

KM: Conceptualization, Data curation, Formal analysis, Investigation, Methodology, Visualization, Writing – original draft, Writing – review & editing. JH: Conceptualization, Data curation, Funding acquisition, Investigation, Methodology, Project administration, Resources, Supervision, Validation, Visualization, Writing – review & editing. CB: Conceptualization, Data curation, Funding acquisition, Methodology, Project administration, Resources, Supervision, Writing – review & editing. DR: Visualization, Writing – review & editing. EV: Visualization, Writing – review & editing, Data curation, Formal analysis, Investigation, Methodology, Writing – original draft. MS: Conceptualization, Data curation, Funding acquisition, Investigation, Methodology, Project administration, Supervision, Validation, Visualization, Writing – review & editing.
