# Supplementary material for: From learned value to sustained bias: how reward conditioning changes attentional priority
Source: Front Hum Neurosci. 2024 Apr 3;18:1354142. doi: 10.3389/fnhum.2024.1354142 (PMC11059963; doi:10.3389/fnhum.2024.1354142)
Supplement: Supplementary file 1 [file Data_Sheet_1.docx]

Supplementary Material

**FROM LEARNED VALUE TO SUSTAINED BIAS: HOW REWARD CONDITIONING CHANGES ATTENTIONAL PRIORITY**

**Kristin N. Meyer*, Joseph B. Hopfinger, Charlotte A. Boettiger, Donita L. Robinson, Elena Vidrascu, Margaret A. Sheridan**

*** Correspondence:** Kristin N. Meyer: Kristin.Meyer1@va.gov

**Behavioral Results for Reaction Time and Accuracy Separately**

# Behavioral Results for Reaction Time and Accuracy Separately

Because attentional orienting can affect both accuracy and reaction times (RT), we chose to analyze inverse efficiency (IE), which can account for speed/accuracy tradeoffs, in our main analyses. To provide additional analyses to compare to previous studies that have used only accuracy or RT, here we provide results from the reward training and cueing task separately for reaction time and accuracy.

## Reward Training

To assess a reward-effect in training, a paired t-test with reward status (rewarded, unrewarded) as a within-subjects factor was implemented on RT and accuracy for both training sessions. Session 1 results indicated that participants were faster on trials with the rewarded color than the unrewarded color in the initial training, *t*(26) = 10.54, *p* < .001, and participants were more accurate to the rewarded relative to unrewarded color *t*(26) = 6.23, *p* < .001. In session 2, the pattern of results was the same, such that participants showed faster RTs [*t*(26) = 10.54, *p* < .001] and higher accuracy [*t*(26) = 6.38, *p* < .001] for the rewarded relative to unrewarded color.

To explore whether the reward effect changed between the training sessions, a 2x2 repeated-measures ANOVA with reward status (rewarded, unrewarded) and session as within-subjects factors was implemented on RT, accuracy, and IE. With regards to reaction time, there was a main effect of reward type, *F*(1, 26) = 146.06, *p* < .001, such that participants were faster to the rewarded than the unrewarded color (Supplementary Table S1). There was also a main effect of session, *F*(1, 26) = 9.02, *p* = .006, wherein participants’ RTs were slower in Session 2 than in Session 1. These main effects were not qualified by a reward by session interaction, *p* = .20. For accuracy, results revealed a main effect of reward type *F*(1, 26) = 61.53, *p* < .001, which was qualified by a reward by session interaction *F*(1, 26) = 5.11, *p* = .03, (Supplementary Table S1). This revealed that participants were more accurate for the rewarded relative to unrewarded colors across both sessions, but the effect of reward on accuracy was larger for Session 2 than Session 1. There was no change in overall accuracy between sessions (*p* = .15).

**1.2 Attention Cueing Paradigm**

It was anticipated that, consistent with value-driven attentional bias, participants would be increasingly worse across valid PR, neutral baseline, and invalid PR trials. To test this linear-orienting effect, a repeated-measures ANOVA was conducted for performance on PR trials with the within-subjects factors of trial type (invalid, baseline, valid) for both RT and accuracy. Results indicated there was an effect of trial type on RT, *F*(2, 52) = 12.22, *p* < .001, *η*^2^ = .320. Within-subjects contrasts confirmed that the effect of trial type on RT was linear, such that performance followed the expected pattern across trial types, *F*(1, 26) = 16.23, *p* < .001, *η*^2^ = .384, Supplementary Figure S1. Similar effects of reward on accuracy were revealed by a significant effect of trial type, *F*(1.55, 40.40) = 4.49, *p* = .024, *η*^2^ = .147. Within-subjects contrasts confirmed that the effect of trial type on accuracy was linear, such that performance followed the expected pattern across trial types, *F*(1, 26) = 6.13, *p* = .020, *η*^2^ = .191, Supplementary Figure S1. Separate paired t-tests examined the specific effects of capture (PR valid relative to neutral baseline) and disengagement (PR invalid relative to neutral) on RT. Results indicated a significant effect of capture, *t*(26) = 2.28, *p* = .031, but no effect of disengagement (*p* = .197) on RT.

To examine whether the orienting effect is value-specific, we tested whether an orienting effect was exhibited for PU cues, which contained the color that was the unrewarded target color in the training phase. A similar repeated-measures ANOVA with the within-subjects factor of trial type (invalid, baseline, valid) was implemented for PU trials. There was no effect of trial type on RT (M_Valid_ = 512 ms, SD_Valid_ = 34 ms, M_Invalid_ = 513 ms, SD_Valid_ = 35 ms, *p* = .893) or accuracy (M_Valid_ = 0.93, SD_Valid_ = 0.08, M_Invalid_ = 0.93, SD_Valid_ = 0.07, *p* = .734). Because there was no evidence of attentional bias, components of attentional bias were not probed.

# fMRIprep Description

Results included in this manuscript come from preprocessing performed using *fMRIPrep* 1.3.2 (Esteban et al., 2019, 2018), which is based on *Nipype* 1.1.9 (Gorgolewski et al., 2011, 2018).

## Anatomical Data Preprocessing

Each subject’s T1-weighted (T1w) image was corrected for intensity non-uniformity (INU) with N4BiasFieldCorrection (Tustison et al., 2010), distributed with ANTs 2.2.0 (Avants, Epstein, Grossman, & Gee, 2008). The T1w-reference was then skull-stripped with a *Nipype* implementation of the antsBrainExtraction.sh workflow (from ANTs), using OASIS30ANTs as a target template. A T1w-reference map was computed after registration of 2 T1w images (after INU-correction) using mri_robust_template (FreeSurfer 6.0.1, Dale, Fischl, & Sereno, 1999). Brain surfaces were reconstructed using recon-all (FreeSurfer 6.0.1, Dale et al., 1999), and the brain mask estimated previously was refined with a custom variation of the method to reconcile ANTs-derived and FreeSurfer-derived segmentations of the cortical gray-matter of Mindboggle (Klein et al., 2017). Spatial normalization to the *ICBM 152 Nonlinear Asymmetrical template version 2009c* (Fonov, Evans, McKinstry, Almli, & Collins, 2009) was performed through nonlinear registration with antsRegistration (ANTs 2.2.0), using brain-extracted versions of both T1w volume and template. Brain tissue segmentation of cerebrospinal fluid (CSF), white-matter (WM), and gray-matter (GM) was performed on the brain-extracted T1w using fast (FSL 5.0.9, Zhang, Brady, & Smith, 2001).

## Functional Data Preprocessing

For each of the 5 BOLD runs per subject (2 runs of reward conditioning task; 3 runs of cueing task), the following steps were performed. First, a reference volume and its skull-stripped version were generated using a custom methodology of *fMRIPrep*. A deformation field to correct for susceptibility distortions was estimated based on extracting the first 3 volumes of task data collected in the AP phase encoding direction and the first 3 volumes of task data collected in the PA phase encoding direction, using 3dQwarp (Cox & Hyde, 1997) (AFNI 20160207). Based on the estimated susceptibility distortion, an unwarped BOLD reference was calculated for a more accurate co-registration with the anatomical reference. The BOLD reference was then co-registered to the T1w reference using bbregister (FreeSurfer), which implements boundary-based registration (Greve & Fischl, 2009). Co-registration was configured with nine degrees of freedom to account for distortions remaining in the BOLD reference. Head-motion parameters with respect to the BOLD reference (transformation matrices, and six corresponding rotation and translation parameters) are estimated before any spatiotemporal filtering using mcflirt (FSL 5.0.9, Jenkinson, Bannister, Brady, & Smith, 2002). BOLD runs were slice-time corrected using 3dTshift from AFNI 20160207 (Cox & Hyde, 1997). The BOLD time-series (including slice-timing correction when applied) were resampled to the *fsaverage5* space and onto their original, native space by applying a single, composite transform to correct for head-motion and susceptibility distortions. Thes latter resampled BOLD time-series will be referred to as *preprocessed BOLD in original space*, or just *preprocessed BOLD*. The BOLD time-series were then resampled to MNI152NLin2009cAsym standard space, generating a *preprocessed BOLD run in MNI152NLin2009cAsym space*. First, a reference volume and its skull-stripped version were generated using a custom methodology of *fMRIPrep*. Several confounding time-series were calculated based on the *preprocessed BOLD*: framewise displacement (FD), DVARS and three region-wise global signals. FD and DVARS are calculated for each functional run, both using their implementations in *Nipype* (following the definitions by Power et al., 2014). Additionally, a set of physiological regressors were extracted to allow for component-based noise correction (*CompCor*, Behzadi et al. 2007). Principal components are estimated after high-pass filtering the *preprocessed BOLD* time-series (using a discrete cosine filter with 128s cut-off) for the anatomical *CompCor* variant (aCompCor). A subcortical mask is obtained by heavily eroding the brain mask, which ensures it does not include cortical GM regions. For aCompCor, six components are calculated within the intersection of the aforementioned mask and the union of CSF and WM masks calculated in T1w space, after their projection to the native space of each functional run (using the inverse BOLD-to-T1w transformation). The head-motion estimates calculated in the correction step were also placed within the corresponding confounds file. All resamplings can be performed with *a single interpolation step* by composing all the pertinent transformations (i.e. head-motion transform matrices, susceptibility distortion correction when available, and co-registrations to anatomical and template spaces). Gridded (volumetric) resamplings were performed using antsApplyTransforms (ANTs), configured with Lanczos interpolation to minimize the smoothing effects of other kernels (Lanczos, 1964). Non-gridded (surface) resamplings were performed using mri_vol2surf (FreeSurfer). Many internal operations of *fMRIPrep* use *Nilearn* 0.5.0 (Abraham et al., 2014), mostly within the functional processing workflow. For more details of the pipeline, see [the section corresponding to workflows in *fMRIPrep’s* documentation](https://fmriprep.readthedocs.io/en/latest/workflows.html) (https://fmriprep.readthedocs.io/en/latest/workflows.html).

**Copyright Waver**

The above boilerplate text was automatically generated by fMRIPrep with the express intention that users should copy and paste this text into their manuscripts unchanged. It is released under the [CC0](https://creativecommons.org/publicdomain/zero/1.0/) license.

# Supplementary Figures

**A.**


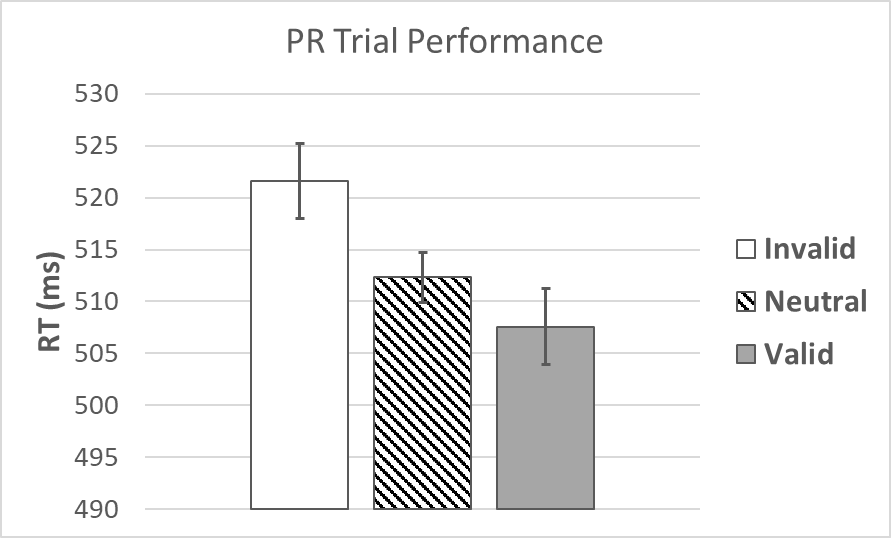

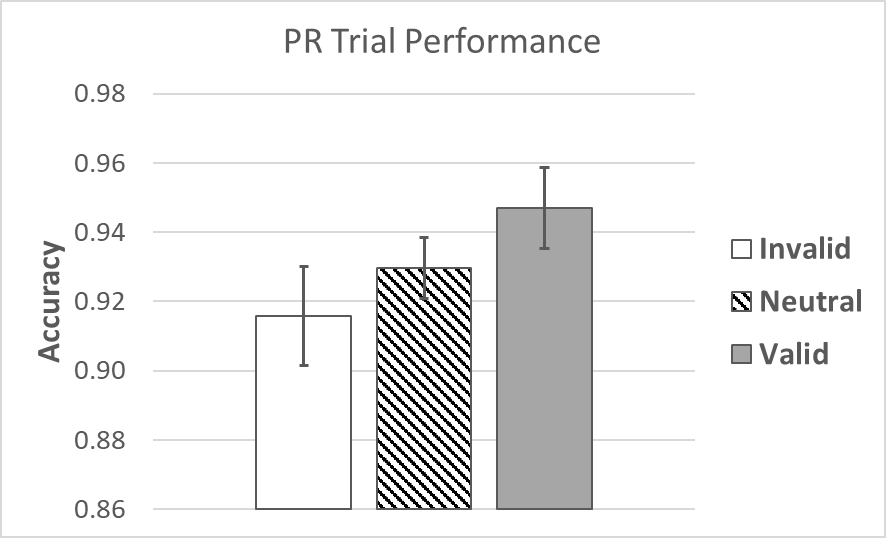


**B.**

**Supplementary Figure S1.** Orienting effects to PR cues on the cueing task for (A) RT and (B) Accuracy. A significant linear orienting effect was found for PR trials on all performance metrics. Error bars are within subjects 95% confidence intervals (Cousineau, 2005).

# Supplementary Tables

| **Supplementary Table S1.** Effect of reward on performance during the reward conditioning task. | | | | | | |
| --- | --- | --- | --- | --- | --- | --- |
|  | **RT** Mean (SD) | | **Accuracy**  Mean (SD) | | **Inverse Efficiency**  Mean (SD) | |
|  | **Rewarded** | **Unrewarded** | **Rewarded** | **Unrewarded** | **Rewarded** | **Unrewarded** |
| **S1** | 553 (37) | 597 (31) | 0.85 (0.07) | 0.77 (0.09) | 652 (70) | 788 (118) |
| **S2** | 569 (35) | 618 (36) | 0.90 (0.08) | 0.77 (0.13) | 637 (99) | 839 (227) |

SD = Standard Deviation. All means and SD report reaction times in milliseconds.

| **Supplementary Table 2.** Whole brain results during reward conditioning task for rewarded targets > unrewarded targets. Negative values for X coordinates indicate left hemisphere, while positive X coordinate values correspond to right hemisphere. | | | | | | |
| --- | --- | --- | --- | --- | --- | --- |
|  |  | **Local Maxima Coordinates** | | |  |  |
| **Cluster** | **Size (Voxels)** | **x** | **y** | **z** | **Z-score** | **Region** |
| 1 | 2553 | 34 | -88 | -12 | 5.78 | Lateral Occipital Cortex |
| 2 | 1841 | -16 | -100 | -10 | 5.52 | Occipital Pole |
| 3 | 1269 | 36 | -60 | 50 | 4.64 | Lateral Occipital Cortex/Superior Parietal Lobule |
| 4 | 598 | 48 | 10 | 32 | 4.74 | MFG/IFG |
| 5 | 587 | 2 | 32 | 52 | 4.75 | Superior Frontal Gyrus/ACC |
| 6 | 306 | -32 | -66 | 48 | 4.32 | Lateral Occipital Cortex |
| 7 | 295 | 36 | 18 | -12 | 3.79 | Anterior Insula |
| 8 | 177 | -34 | 16 | -4 | 3.57 | Anterior Insula |
| 9 | 176 | -30 | -66 | -26 | 3.97 | Cerebellum |
| 10 | 161 | 44 | 58 | 0 | 4.38 | Ventrolateral Prefrontal Cortex |
| 11 | 123 | -26 | -72 | -46 | 3.31 | Cerebellum |
| 12 | 116 | 8 | 18 | 4 | 4.14 | Right Caudate |
| 13 | 106 | 3 | -44 | -54 | 3.00 | Superior Parietal Lobule |
| 14 | 102 | 2 | -94 | 10 | 4.11 | Occipital Pole |
| 15 | 97 | -2 | -32 | 24 | 3.58 | PCC |

ACC = anterior cingulate cortex. PCC = posterior cingulate cortex. MFG = middle frontal gyrus. IFG = inferior frontal gyrus. Anatomical regions defined according to Harvard Oxford Atlas in FSL.

Results reported with *p* < .05, FWE corrected.

|  | | | | | | | | |
| --- | --- | --- | --- | --- | --- | --- | --- | --- |
| **Supplementary Table 3.** Whole brain results for group maps for Testing/Cueing task. | | | | | | | | |
|  |  |  | **Local Maxima Coordinates** | | |  |  | |
| **Contrast** | **Cluster** | **Size (Voxels)** | **x** | **y** | **z** | **Z-score** | | **Region** |
| PR > Neutral | 1 | 2284 | 22 | -16 | 76 | 4.66 | Precentral Gyrus | |
|  | 2 | 1455 | -24 | -72 | -16 | 4.49 | Left Occipital Fusiform gyrus | |
|  |  |  | -44 | -76 | -10 | 3.89 | Left LOC | |
|  | 3 | 959 | 44 | -60 | -18 | 4.00 | Right Occipital Fusiform Gyrus | |
|  |  |  | 38 | -78 | 2 | 3.69 | Right LOC | |
|  | 4 | 258 | 8 | -12 | 58 | 4.08 | SMA | |
|  | 5 | 140 | -60 | -2 | 14 | 3.54 | Precentral Gyrus | |
|  | 6 | 135 | 32 | -58 | 50 | 4.08 | Right SPL | |
|  |  |  |  |  |  |  |  | |
| PU > Neutral | 1 | 718 | 44 | -14 | 54 | 4.92 | Precentral Gyrus | |
|  | 2 | 146 | -54 | 28 | -6 | 3.63 | Frontal Orbital Cortex | |
|  | 3 | 136 | 6 | -14 | 60 | 3.64 | SMA | |
| PR > PU | 1 | 84 | 54 | -22 | 36 | 3.61 | Postcentral Gyrus | |
| PU > PR | 1 | 477 | -6 | 46 | 20 | 3.62 | vmPFC | |
| Neutral > PR | 1 | 375 | -2 | 58 | 2 | 3.76 | vmPFC | |
|  | 2 | 275 | 26 | 22 | 50 | 4.44 | Middle Frontal Gyrus | |
|  | 3 | 132 | 54 | -72 | 32 | 3.62 | Right LOC | |
|  | 4 | 90 | 56 | -54 | 48 | 3.48 | Angular Gyrus | |
| Neutral > PU | - | - | - | - | - | - | No voxels | |

PR = previously rewarded. PU = previously unrewarded. LOC = lateral occipital cortex. SPL = superior parietal lobule. SMA = supplementary motor cortex. vmPFC = ventromedial prefrontal cortex. Anatomical regions defined according to Harvard Oxford Atlas in FSL.

Results reported with *p* < .05, FWE corrected.

| **Supplementary Table 4.** Whole brain results for group maps, for Testing/Cuing Task by Validity. | | | | | | | |
| --- | --- | --- | --- | --- | --- | --- | --- |
|  |  |  | **Local Maxima Coordinates** | | |  |  |
| **Contrast** | **Cluster** | **Size (Voxels)** | **x** | **y** | **z** | **Z-score** | **Region** |
| PR Invalid > PU Invalid | 1 | 146 | 54 | -22 | 36 | 3.86 | Supramarginal gyrus |
|  | 2 | 137 | 36 | -60 | 48 | 3.56 | LOC |
|  | 3 | 109 | -32 | -66 | -20 | 3.84 | Occipital Fusiform gyrus |
|  | 4 | 102 | 36 | -46 | 46 | 3.59 | SPL |
| PR Invalid > Neutral | 1 | 1837 | 42 | -16 | 54 | 4.61 | Precentral gyrus |
|  | 2 | 806 | -48 | -58 | -10 | 4.08 | Left LOC |
|  | 3 | 472 | 44 | -62 | -14 | 3.7 | Right LOC |
|  | 4 | 219 | -6 | 6 | 56 | 3.68 | SMA |
|  | 5 | 179 | 32 | -56 | 58 | 3.67 | Right SPL |
|  | 6 | 151 | -28 | -74 | 30 | 3.59 | Left LOC |
| PR Valid > Neutral | 1 | 669 | 32 | -24 | 46 | 3.82 | Precentral gyrus |
|  | 2 | 372 | 46 | -52 | -24 | 4.05 | Temporal Occipital Fusiform gyrus |
|  |  |  | 40 | -72 | -18 | 3.72 | LOC |
|  | 3 | 357 | 16 | -76 | 30 | 4.4 | Precuneus cortex |
|  |  |  | 28 | -82 | 36 | 3.32 | Intraparietal Sulcus |
|  | 4 | 316 | -12 | -80 | 28 | 4.02 | Precuneus cortex |
|  |  |  | -2 | -90 | 26 | 3.47 | Occipital Pole |
|  | 5 | 313 | 56 | -10 | 14 | 4.85 | Central Opercular cortex |
|  | 6 | 235 | -62 | -18 | 8 | 4.07 | Superior Temporal gyrus |
|  | 7 | 163 | -30 | -82 | 26 | 3.47 | LOC |
|  | 8 | 163 | -36 | -54 | -16 | 4.26 | Occipital Fusiform gyrus |
|  | 9 | 147 | -14 | -82 | -4 | 3.8 |  |
|  | 10 | 90 | -54 | -74 | 8 | 3.43 | LOC |
|  | 11 | 88 | -2 | -14 | 60 | 3.85 | SMA |

PR = previously rewarded. PU = previously unrewarded. LOC = lateral occipital cortex. SPL = superior parietal lobule. SMA = supplementary motor cortex. Anatomical regions defined according to Harvard Oxford Atlas in FSL.

Results reported with *p* < .05, FWE corrected.

| **Supplementary Table 5.** Whole brain results for group maps for testing/cueing task as predicted by striatal activity during reward training phase. | | | | | | | |
| --- | --- | --- | --- | --- | --- | --- | --- |
|  |  |  | **Local Maxima Coordinates** | | |  |  |
| **Contrast** | **Cluster** | **Size (Voxels)** | **x** | **y** | **z** | **Z-score** | **Region** |
| PR > Neutral | 1 | 1997 | -38 | -42 | 38 | 4.65 | Supramarginal Gyrus |
|  |  |  | -24 | -78 | 52 | 4.43 | Left LOC |
|  |  |  | 10 | -68 | 58 | 4.33 | Right LOC |
|  | 2 | 413 | -8 | -92 | -18 | 3.96 | Occipital Pole |
|  | 3 | 239 | -48 | -14 | 12 | 4.04 | Central Opercular Cortex |
|  | 4 | 238 | -12 | -98 | 30 | 4.76 | Occipital Pole |
|  | 5 | 237 | -48 | -82 | 20 | 4.01 | Left LOC |
|  | 6 | 169 | -58 | -70 | -8 | 3.98 | Left LOC |
| PR Valid > Neutral | 1 | 3126 | -12 | -98 | 30 | 4.53 | Occipital pole |
|  |  |  | 10 | -68 | 58 | 4.36 | LOC |
|  | 2 | 263 | -54 | -8 | 24 | 3.92 | Postcentral Gyrus |
|  | 3 | 188 | 4 | -82 | -24 | 3.67 | Cerebellum |
|  |  |  | 26 | -90 | -18 | 3.4 | Occipital fusiform gyrus |
|  | 4 | 141 | 68 | -10 | 20 | 3.82 | Postcentral Gyrus |
|  | 5 | 137 | 58 | -48 | 16 | 4.01 | Angular Gyrus |
| PR Invalid > Neutral | 1 | 161 | 4 | -50 | 70 | 3.96 | Precuneus Cortex |
|  | 2 | 89 | 2 | 8 | 48 | 3.59 | ACC |
| PR Invalid > PU Invalid | 1 | 183 | 2 | 12 | 42 | 3.94 | ACC |
| PU > PR | 1 | 115 | -4 | 42 | 14 | 3.32 | vmPFC |

PR = previously rewarded. PU = previously unrewarded. LOC = lateral occipital cortex. ACC = anterior cingulate cortex. vmPFC = ventromedial prefrontal cortex. Anatomical regions defined according to Harvard Oxford Atlas in FSL.

Results reported with *p* < .05, FWE corrected.
